# Analysis of Dynamics and Diversity of Microbial Community during Production of Germinated Brown Rice

**DOI:** 10.3390/foods12040755

**Published:** 2023-02-09

**Authors:** Gaoji Yang, Juanjuan Xu, Yuanmei Xu, Rui Li, Shaojin Wang

**Affiliations:** 1 College of Mechanical and Electronic Engineering, Northwest A & F University, Xianyang 712100, China; 2 Department of Biological Systems Engineering, Washington State University, 213 L.J. Smith Hall, Pullman, WA 99164-6120, USA

**Keywords:** brown rice, sprout process, microbiome, high-throughput sequencing, pathogens

## Abstract

Sprouts may be contaminated with different pathogenic and spoilage microorganisms, which lead far too easily to foodborne outbreaks. The elucidations of microbial profiles in germinated brown rice (BR) are important, but the changes in the microbial composition during germination are unknown. This study aimed to investigate the microbiota composition and to monitor the dominant microbial dynamics in BR during germination using both culture-independent and -dependent methods. BR samples (HLJ2 and HN) were collected from each stage of the germination processing. The populations of microbes (total viable counts, yeast/mold counts, *Bacillus cereus*, and *Enterobacteriaceae*) of two BR cultivars increased markedly with the prolongation of the germination time. High-throughput sequencing (HTS) showed that the germination process significantly influenced the microbial composition and reduced the microbial diversity. Similar microbial communities were observed between the HLJ2 and the HN samples, but with different microbial richness. The bacterial and fungal alpha diversity achieved the maximum for ungerminated samples and declined significantly after soaking and germination. During germination, *Pantoea*, *Bacillus*, and *Cronobacter* were the dominant bacterial genera, but *Aspergillus*, *Rhizopus*, and *Coniothyrium* dominated for the fungi in the BR samples. The predominance of harmful and spoilage microorganisms in BR during germination is mainly from contaminated seeds, which highlights the potential risk of foodborne illness from sprouted BR products. The results provide new insight into the microbiome dynamics of BR and may help to establish effective decontamination measures against pathogenic microorganisms during sprout production.

## 1. Introduction

Brown rice (BR), a processed product of rice only after shucking, is considered as whole grain and has attracted widespread attention due to its rich nutritional components [1]. Germination is an effective process to improve the nutritional quality, digestibility, and flavor of cereals [2]. Currently, germinated brown rice (GBR) has gained significant attention due to the use of a processing approach to enhance the food quality and potential health-promoting functions [3]. However, due to the high nutritional values in the sprouts and the suitable environment in the BR germination process, the rice grain-associated microorganisms can readily proliferate on the surface of BR [4,5]. It has also been shown that the microbial community of the grains influences the quality of the sprouts and may also cause a risk of foodborne illness [6,7,8]. More importantly, there are limited reports on the systematic elucidation of the microbial profile during the germination of BR. 

A previous study reported by Kim et al. [4] determined the microbial counts of total mesophilic aerobic bacteria, lactic acid bacteria (LAB), and *Enterobacteriaceae* during BR germination processing. Bourneow and Toontam [6] indicated that the aerobic mesophilic bacteria, yeasts, and molds were found in the initial stage of BR germination, but the LAB were found in the late phase of the process. These microbial contaminations of sprouted products mainly derived from the initial microbes of the seeds. The microflora of cereal grains or seeds depends on the stage of processing or the environmental conditions, including the pre-harvest practices, the decontamination treatment, and the seed storage [7,9]. The common bacteria in cereal grains are mainly *Pseudomonadaceae*, *Micrococcaceae*, *Lactobacillaceae*, and *Bacillaceae* [10], which are generally non-pathogenic. Nevertheless, pathogens such as *Salmonella*, *Cronobacter sakazakii*, and *Bacillus cereus* can also contaminate the grains. BR was shown to be contaminated with total mesophilic bacteria, LAB, total coliforms, enterococci, yeasts, and molds at the levels of 7.45, 3.84, 3.08, 1.30, and 4.41 log colony-forming units (CFU)/g, respectively [11]. Additionally, microbiological surveys have shown the association of a variety of foodborne pathogens in sprouts [7]. Dechet et al. [12] reported 33 foodborne outbreaks in the United States from 1998 to 2010 due to the consumption of seed and bean sprouts, which affected 1330 persons. A study has also shown that *Salmonella* was the leading pathogen of foodborne outbreaks in the European Union from 2004 to 2012 [13]. The microbial contamination may result in a fermented odor or another unpleasant odor in GBR [6]. In addition, the growth of microorganisms may consume some macromolecular substances in sprouts and alter the enzyme activity, which adversely affects the formation of the nutrients [14].

Recently, many studies have used conventional culture-dependent methods to investigate the microbial diversity [4,6]. However, these methods may not completely reflect the microflora information since several strains are difficult to isolate and cultivate due to the specific culture conditions, such as a certain temperature, moisture, oxygen concentration, and media [8]. High-throughput sequencing (HTS) based on culture-independent techniques is now commonly employed to analyze the microbial community and dynamics in food-based ecosystems, such as cereal grain fermentation [15,16], sprout storage [17], and pretreatment on grains [18,19]. Indeed, an HTS approach was employed to access the microbial composition in several sprouts, such as alfalfa, mung beans, radish, and barley [8,17,20]. However, systematic changes in the microbiota, including the bacterial and fungal communities during the germination of BR, have not been reported. Consequently, the objective of this study was to determine the microbial diversity changes in the natural microbiota in the seeds at different BR germination stages and to reveal the dynamics of the dominant microbiota from seed to sprout. Analysis of the microbial community and the dominant genera dynamics during the germination of BR can provide the microbial profiles of the pathogens and spoilage microbes that need to be controlled. Elucidations of microbiota changes (including microbial diversity and the dynamics of the dominant genera) may facilitate the determination of effective decontamination measures during GBR production.

## 2. Materials and Methods

### 2.1. Brown Rice Samples and Preparation

Thirteen different BR samples were purchased from the major production provinces (Anhui, Guangdong, Guangxi, Hubei, Heilongjiang, Hunan, Jiangsu, Jiangxi, Liaoning, and Sichuan) in China. The initial moisture content (MC) of the BR samples was measured using a moisture analyzer (HE53, Mettler-Toledo, Shanghai, China). The BR samples were stored at 4 °C until used for the experiments.

### 2.2. Germination of Brown Rice

The germination process was conducted according to Moongngarm and Saetung [21], with some modifications. The BR kernels (100 g) were soaked in sterilized distilled water at a ratio of 1:5 (*w*/*v*) at 30 °C for 12 h, and the used water was replaced every 6 h. After washing, the BR grains were drained and spread in Petri dishes (90 mm inner diameter), covered with two wet layers of sterilized filter paper, and germinated in a constant temperature and humidity chamber (HWS-150, Senxin Experimental Instrument Co., Ltd., Shanghai, China) at 30 °C for 48 h. During the germination period, the grains were sprayed with distilled water every 6 h to maintain humidity. Samples were collected at 12 h intervals. Untreated BR grains were used as the ungerminated samples (control) in this study.

### 2.3. Microbiological Determination

For each ungerminated and germinated BR, samples (3 g) were taken and transferred into sterile tubes containing 27 mL sterile saline (0.85% NaCl) and mixed completely by vortex (CY-30S, Yueqia Experimental Instrument Co., Ltd., Shanghai, China). After serial dilution (1:10), 0.1 mL of diluent was spread on (1) plate count agar (Luqiao Co. Ltd., Beijing, China) to enumerate the total viable counts (TVC) and incubated at 37 °C for 48 h; (2) rose bengal medium (Luqiao Co. Ltd., Beijing, China) and incubated at 28 °C for 72 h to enumerate the yeast and mold counts; (3) mannitol–egg yolk–polymyxin agar (Hopebio, Qingdao, China) to enumerate the *B. cereus* and incubated at 37 °C for 24 h; and (4) violet red bile glucose agar (Luqiao Co. Ltd., Beijing, China) and placed at 37 °C for 24 h to determine the *Enterobacteriaceae* counts. The counted and reported colonies on the plate of each sample were converted into log_10_ CFU/g.

### 2.4. Microbial Diversity Analysis

#### 2.4.1. DNA Extraction and PCR Amplification

Ungerminated and germinated BR grains (10 g) were added into sterile 50 mL centrifuge tubes containing the maximum recovery diluent and vigorously shaken for 15 min. The solution was centrifuged (8000 rpm, 10 min, 4 °C), and the pellet was washed three times and resuspended in sterile saline. The final pellet was stored at −80 °C until used, and then, it was used for the extraction of microbial genomic DNA using a Power Water DNA Isolation Kit (MO BIO Laboratories, Carlsbad, CA, USA) according to the manufacturer’s instructions. The DNA concentration was quantified with a Qubit Fluorometer by using the Qubit dsDNA BR Assay kit (Invitrogen, Carlsbad, CA, USA), and the purity was checked by running aliquot on a 1% agarose gel.

Variable regions (V3-V4) of the bacterial 16S rRNA gene were amplified with the primer pairs 341F (5′-ACTCCTACGGGAGGCAGCAG-3′) and 806R (5′-GGACTACHVGGGTWTCTAAT-3′). The fungal ITS2 of the Internal Transcribed Spacer (ITS) region was amplified with the primer pairs ITS3F (5′-GCATCGATGAAGAACGCAGC-3′) and ITS4R (5′-TCCTCCGCTTATTGATATGC-3′). The PCR amplification was performed as follows: 94 °C for 3 min, 30 cycles of 94 °C for 30 s, 56 °C (16S) or 58 °C (ITS) for 45 s, 72 °C for 45 s, and a final extension at 72 °C for 10 min. The PCR products were purified with AmpureXP beads and eluted in elution buffer. In the PCR amplification, the complete reaction mixtures were set up, and no template controls in the reactions were used.

#### 2.4.2. Sequencing and Bioinformatics Processing

The purified amplicons were pooled in equimolar and paired-end sequenced (2 × 300) on an Illumina MiSeq platform (Illumina, San Diego, CA, USA) using a TruSeq™ sample preparation kit (Illumina Inc., San Diego, CA, USA) and conducted by BGI Genomics, Shenzhen, China. The sequence data obtained from the high-throughput analysis in this study were deposited in the NCBI BioProject PRJNA896727 (Bacteria) and PRJNA896764 (Fungi).

Raw reads were filtered to remove adaptors and low-quality and ambiguous bases, and then, the paired-end reads were added to tags by the FLASH reads program (v1.2.11) [22]. The tags were clustered into operational taxonomic units (OTUs) with a cutoff value of 97% using UPARSE software (v7. 0.1090, http://drive5.com/uparse/, accessed on 4 October 2021) [23], and the chimera sequences were compared with the Gold database (v20110519) or UNITE (v20170628) using UCHIME (v4.2.40) [24]. Then, the OTU representative sequences were taxonomically classified using Ribosomal Database Project (RDP) Classifier v.2.2, with a minimum confidence threshold of 0.6, and trained on the Greengenes database v201305 by QIIME v1.8.0 [25]. USEARCH_global was used to compare all the tags back to the OTUs to obtain the OTU abundance statistics table of each sample [26]. 

The alpha and beta diversity were estimated by MOTHUR (v1.31.2) [27] and QIIME (v1.8.0) at the OTU level, respectively. The sample cluster was conducted by QIIME (v1.8.0) based on UPGMA. The bar plot and heatmap of the different classification levels were plotted with R package v3.4.1 and R package “gplots”, respectively. Principal co-ordinates analysis (PCoA) was used to determine the difference in bacterial community composition between the samples. The LDA effect size (LEfSe) cluster or LDA analysis was conducted by LEfSe. Based on the species annotation and the abundance of effective OTUs, the functional annotations were obtained based on the Kyoto Encyclopedia of Genes and Genomes (KEGG) pathway using Tax4Fun v1.0. The significant species or functions were determined by R (v3.4.1) based on the Wilcox test or the Kruskal test.

### 2.5. Statistical Analysis

The statistical analyses were performed using SPSS software (Version 24.0; SPSS, Inc, Chicago, IL, USA). The data are presented as the mean ± SD (*n* = 3), and the differences between the means were tested via one-way ANOVA. Individual or replicate samples from each province were analyzed. All experiments were performed in triplicate, and each biological experiment included three technical replicates. A *p* value < 0.05 was considered statistically significant.

## 3. Results

### 3.1. Natural Microbial Counts of BR Samples

The microbiological profiles of 13 BR samples, including the TVC, the yeast/mold counts, and the *B. cereus* and *Enterobacteriaceae* counts, are listed in Table 1. The TVC and yeast/mold counts of 13 samples were in the range of 4.10–5.86 log CFU/g and 3.93–5.33 log CFU/g, respectively. Apart from the LN2 sample, the foodborne pathogen *B. cereus* could be detected in the remaining 12 samples. The *B. cereus* counts detected from the nine BR samples (AH2, GD, GX, HB, HLJ2, HN, JS, LN1, and SC) ranged from 1.54 to 2.59 log CFU/g. The *Enterobacteriaceae* counts in the 13 BR samples varied from 3.09 to 5.22 log CFU/g. There were significant differences (*p* < 0.05) in the counts of the yeast/mold, *B. cereus*, and *Enterobacteriaceae* among the 13 BR samples, and the results indicated that the BR could be easily contaminated by various microbes.

### 3.2. Microbiological Changes during BR Germination

**Figure 1:**
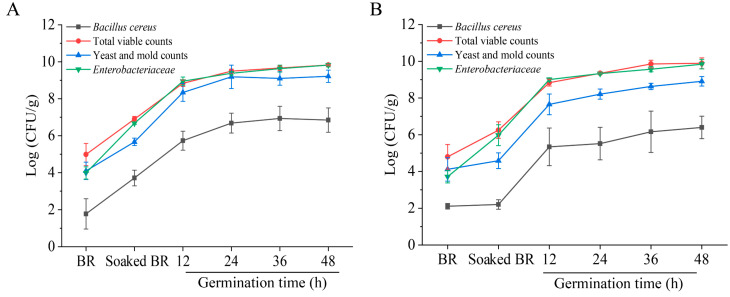
The microbiological profiles of BR samples at various germination times: (**A**) HLJ2; (**B**) HN. BR: untreated BR grains; soaked BR: the BR kernels were soaked in distilled water with a ratio of 1:5 (*w*/*v*) at 30 °C for 12 h.

Two different cultivars of BR samples (Japonica, HLJ2 and Indica, HN) were selected for further study according to the microbial loads and germination rate from 13 samples (0–90.3%). The germination rate of the HLJ2 (73.3%) and HN (90.3%) samples was higher compared to the other samples (<70%) after germination for 48 h. The changes in the microbial enumeration of both samples at different germination times are presented in Figure 1. It was obvious that the TVC, the yeast/mold counts, and the *B. cereus* and *Enterobacteriaceae* numbers of the HLJ2 and HN samples increased significantly during the process of soaking and germination. After soaking for 12 h, the *B. cereus*, TVC, yeast/mold, and *Enterobacteriaceae* counts of HLJ2 significantly increased from 1.77 to 3.71, 4.98 to 6.90, 4.10 to 5.68, and 3.98 to 6.65 log CFU/g, respectively (Figure 1A). However, for the HN sample, there was no significant difference (*p* > 0.05) in the *B. cereus* numbers after soaking (Figure 1B). After germination for 48 h, the *B. cereus*, TVC, yeast/mold, and *Enterobacteriaceae* counts of the HLJ2 samples increased to 6.85, 9.83, 9.21, and 9.82 log CFU/g, respectively. Similarly, for the HN samples, these microbial counts increased to 6.40, 9.89, 8.91, and 9.84 log CFU/g, respectively. These results showed that the populations of microbes rose markedly during the BR germination.

### 3.3. Microbiome Analysis

#### 3.3.1. Community Abundance and Diversity

Effective sequences of 2,132,479 and 2,267,150 were entirely obtained from the 16S rRNA and ITS libraries, respectively. The sequences were clustered in 2311 bacterial OTUs (ranging from 31 to 234) and 3558 fungal OTUs (ranging from 14 to 319) using a 97% similarity threshold. As shown in Supplementary Tables S1 and S2, the Good’s coverage value was more than 99.9% with regard to both the bacterial and the fungal community abundance, demonstrating that the samples had a high coverage rate. The rarefaction curves (Supplementary Figure S1) for the 16S rRNA and ITS gene sequences tended to be saturated, indicating that the sequencing results could truly reflect the microbial profile during BR germination. The trend of the microbial species number under the two BR samples was consistent with the findings on the alpha diversity analysis (Supplementary Tables S1 and S2), assessed using the Sobs, Chao, ACE, Shannon, and Simpson indexes. For the bacteria, the OTUs and the Chao, ACE, and Shannon indexes in the HLJ2 and HN samples decreased during germination. The highest indexes were found in the ungerminated samples (HLJ2 and HN), which were 220, 236, 240, and 3.14 as well as 156, 171, 178, and 3.01, respectively. For the species diversity and richness of fungi, the indexes in the HLJ2 and HN samples decreased during germination. Similarly, these indexes reached the maximum in the ungerminated samples and were 309, 355, 363, and 2.95 as well as 252, 276, 281, and 3.04, respectively. These changes in the four indexes indicated that the richness and diversity decreased during germination, suggesting that a subset of bacteria or fungi became dominant in the BR samples.

Based on the OTU abundance, Venn diagrams (samples < 5) and flower diagrams (samples > 5) were used to show the shared and unique OTUs in each group. The Venn diagrams showed that there were 170 common OTUs for the bacteria and 226 for the fungi in two cultivars (Supplementary Figure S2). The number of unique OTUs for the bacteria was 110 in HLJ2 and 41 in HN; for the fungi, it was 150 in HLJ2 and 108 in HN. For the bacteria, the HLJ2 samples shared 25 OTUs, which were slightly lower than those (30) in the HN samples. The numbers of unique OTUs decreased obviously in the two BR cultivars (Supplementary Figure S3A,B). The ungerminated samples (HLJ2 and HN) had the highest unique OTU (183 and 145) numbers, and the unique OTUs were found to have reduced to a minimum of 0 after germinating for 48 h. For the fungi, the HLJ2 samples shared 42 OTUs, which were higher than those (20) in the HN samples (Supplementary Figure S3C,D). It was also found that the numbers of unique OTUs decreased obviously in the two BR cultivars. These results indicated that new species (except fungi) rarely appeared, and only certain species were found during germination. 

**Figure 2:**
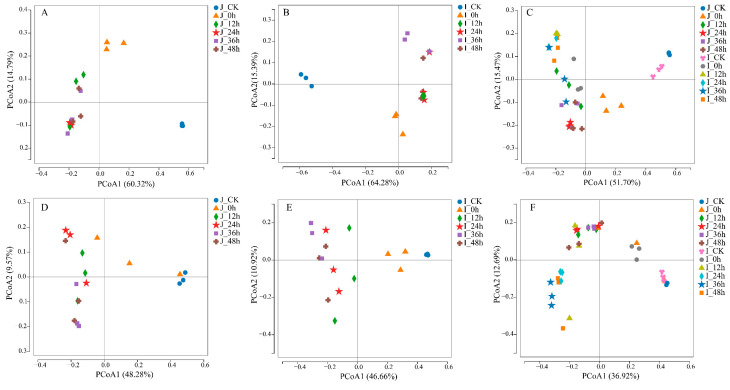
PCoA plot analysis based on unweighted UniFrac metrics for HLJ2 (**A**,**D**), HN (**B**,**E**), and all samples (**C**,**F**) at various germination times; (**A**–**C**) bacteria; (**D**–**F**) fungi. Control: untreated BR grains were used as the ungerminated samples. Samples germinated for 0 h represented the soaked BR. Labels ‘J’ and ‘I’ in legend refer to HLJ2 and HN samples, respectively.

Principal coordinates analysis (PCoA) was used to analyze the data similarities or differences in the bacterial and fungal communities between the samples. There were significant differences in the beta diversity of the bacterial communities in the HLJ2 samples (unweighted UniFrac, R^2^ = 0.7952, *p* = 0.0001) and the HN samples (unweighted UniFrac, R^2^ = 0.8384, *p* = 0.0001) among the different development stages (Figure 2A,B). For the HLJ2 and HN samples, the bacterial communities in the ungerminated and soaked stages were obviously separated, and the other samples in the germinated stages were clustered together. However, significance was confirmed only for the ungerminated BR samples (J_CK and I_CK) using PERMANOVA tests among the samples (*p* < 0.05). For the fungi, the HLJ2 and HN samples exhibited similar clustering and separating trends (except for the soaked samples clustered with the ungerminated samples), suggesting differences in their microbiota after germination (Figure 2C,D). The beta diversity between the two cultivars at each development stage was also compared (Figure 2C,F). The results revealed that the microbial communities between the HLJ2 and the HN samples were not significantly different at each stage (*p* > 0.05). 

Overall, the diversity analysis demonstrated that the structures of the microbial communities at the different germination stages of the two BR cultivars were obviously different. In particular, the germination process had a significant effect on the microbiota in the BR samples. Further analysis of each stage is also required to identify rapid changes in the microbial community structure.

#### 3.3.2. Composition Analysis of Bacterial Community during BR Germination

**Figure 3:**
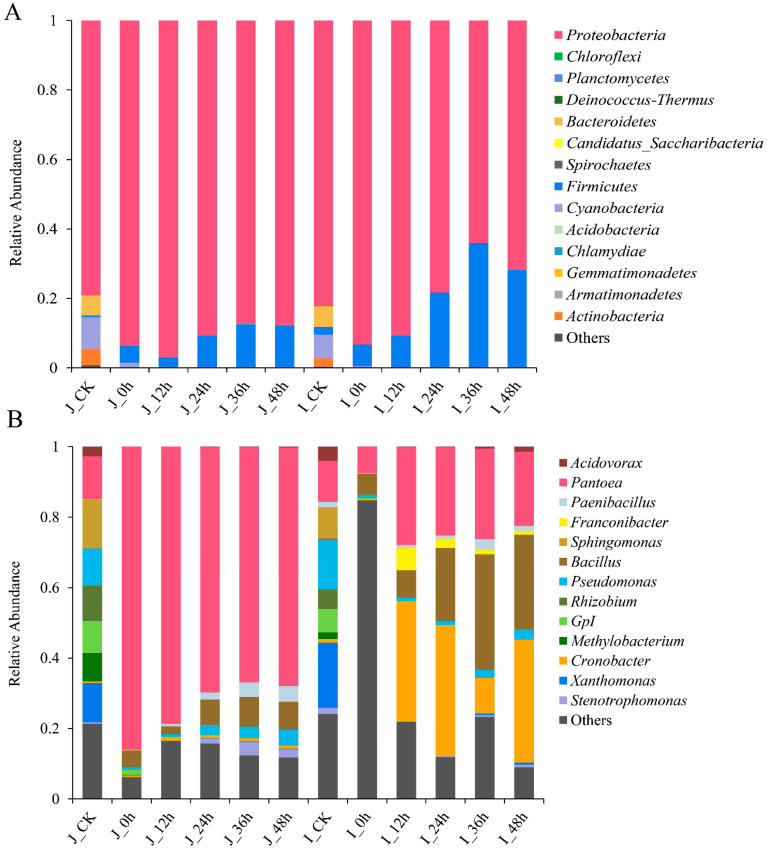
Bacterial community structure variation of HLJ2 and HN samples at different stages. The relative abundance of bacteria at the (**A**) phylum and (**B**) genus levels is shown. Genera of abundance > 0.5% are listed and genera of abundance < 0.5% and unclassified are marked as others. Labels ‘J’ and ‘I’ in legend refer to HLJ2 and HN samples, respectively.

Fourteen bacterial phyla were detected by taxonomical classification in all the samples (Figure 3A), but there were no significant differences between the HLJ2 and the HN samples in terms of the bacterial community composition. For the HLJ2 and the HN samples, *Proteobacteria* (79.14% and 82.18%), *Cyanobacteria* (9.10% and 6.77%), and *Bacteroidetes* (5.78% and 6.00%) were the dominant phyla in the ungerminated stage. However, *Cyanobacteria* and *Bacteroidetes* (<0.001%) nearly disappeared, and *Proteobacteria* (87.69% and 71.79%) and *Firmicutes* (12.18% and 28.20%) became the major phyla after germination for 48 h. 

At the genus level, the bacterial composition of the BR samples was significantly influenced by the germination process (Figure 3B). No significant difference in bacterial composition was observed between the ungerminated HLJ2 and HN samples, while the bacterial communities and relative abundances of the genera were obviously different in the two cultivars during germination processing. A total of 130 genera were detected among all the stages of the HLJ2 samples. The genera with a relative abundance of greater than 5% were *Sphingomonas* (13.78%), *Pantoea* (12.28%), *Xanthomonas* (11.18%), *Pseudomonas* (10.55%), *Rhizobium* (9.99%), *GPI* (9.06%, *GPI* belongs to the *Cyanobacteria* group), and *Methylobacterium* (8.10%), respectively. The abundance of *Pantoea* obviously increased (86.03%) after the soaking process and was reduced to 67.73% at the end of germination stage due to the other species gradually increasing in numbers. *Bacillus* and *Paenibacillus* were also detected to have increased, with the abundances of 7.79% and 4.37% after germination for 48 h. For the HN samples, the top six abundant genera (relative abundance > 5%) included *Xanthomonas* (18.84%), *Pseudomonas* (14.12%), *Pantoea* (11.83%), *Sphingomonas* (9.00%), *GPI* (6.77%), and *Rhizobium* (5.77%), which accounted for 66.33% of all of the genera in the ungerminated samples. Notably, *Cronobacter* was dominant in the HN samples, with an abundance of 34.99% after germination for 48 h, followed by *Bacillus* and *Pantoea* at 26.79% and 21.06%. It was indicated that the major bacterial genera in the BR samples shifted significantly after germination treatment.

#### 3.3.3. Composition Analysis of Fungal Community during BR Germination

**Figure 4:**
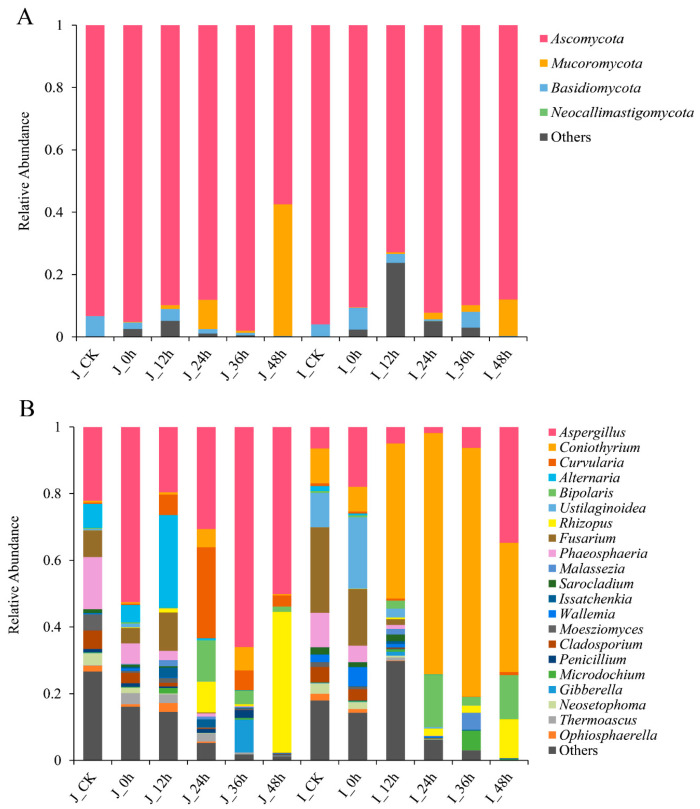
Fungal community structure variation of HLJ2 and HN samples at different stages. The relative abundance of fungi at the (**A**) phylum and (**B**) genus levels is shown. Genera of abundance > 0.5% are listed, and genera of abundance < 0.5% and unclassified are marked as others. Labels ‘J’ and ‘I’ in legend refer to HLJ2 and HN samples, respectively.

The fungal community comprised four phyla, including *Ascomycota*, *Mucoromycota*, *Basidiomycota*, and *Neocallimastigomycota* in the all samples (Figure 4A). *Ascomycota* was always the dominant phylum, with the relative abundance ranging from 57.45 to 98.04% and 72.87 to 95.94% in the HLJ2 and HN samples. During germination, the abundance of *Ascomycota* gradually decreased but that of *Mucoromycota* increased.

At the genus level, a total of 170 fungal genera were detected in all the samples, and 21 genera are shown in Figure 4B. The germination process obviously influenced the fungal composition of the HLJ2 and HN samples. A similar fungal composition was detected between the HLJ2 and the HN samples at the ungerminated stage. However, the differences in the abundance of the fungal communities between the two cultivars were significant during the germination process (*p* < 0.05). There were 18 genera determined in the ungerminated HLJ2 samples, and the top 5 genera were *Aspergillus* (22.09%), *Phaeosphaeria* (15.58%), *Fusarium* (8.06%), *Alternaria* (7.21%), and *Cladosporium* (5.47%). After germination for 48 h, *Aspergillus* and *Rhizopus* were the main groups, with the relative abundances of 50.20% and 42.21%. The relative abundances of the other genera, especially *Alternaria* and *Curvularia*, were also increased by the germination process. Fifteen genera of fungi were detected during the germination process in the HN samples. It was found that the fungal diversity was obviously quite different from that of the HLJ2 samples. For the ungerminated samples, the top five genera were *Fusarium* (25.69%), *Coniothyrium* (10.51%), *Phaeosphaeria* (10.30%), *Ustilaginoidea* (10.24%), and *Aspergillus* (6.44%). During the germination phase, *Coniothyrium* was the main dominant genus. The abundance of *Coniothyrium* increased after germination for 12 h but then decreased at 48 h. In addition, *Aspergillus*, *Bipolaris*, and *Rhizopus* were more abundant after germination, but the abundance of *Fusarium*, *Ustilaginoidea*, and *Phaeosphaeria* decreased (Figure 4B).

#### 3.3.4. Comparison of Microbial Community Structure between HLJ2 and HN Samples

**Figure 5:**
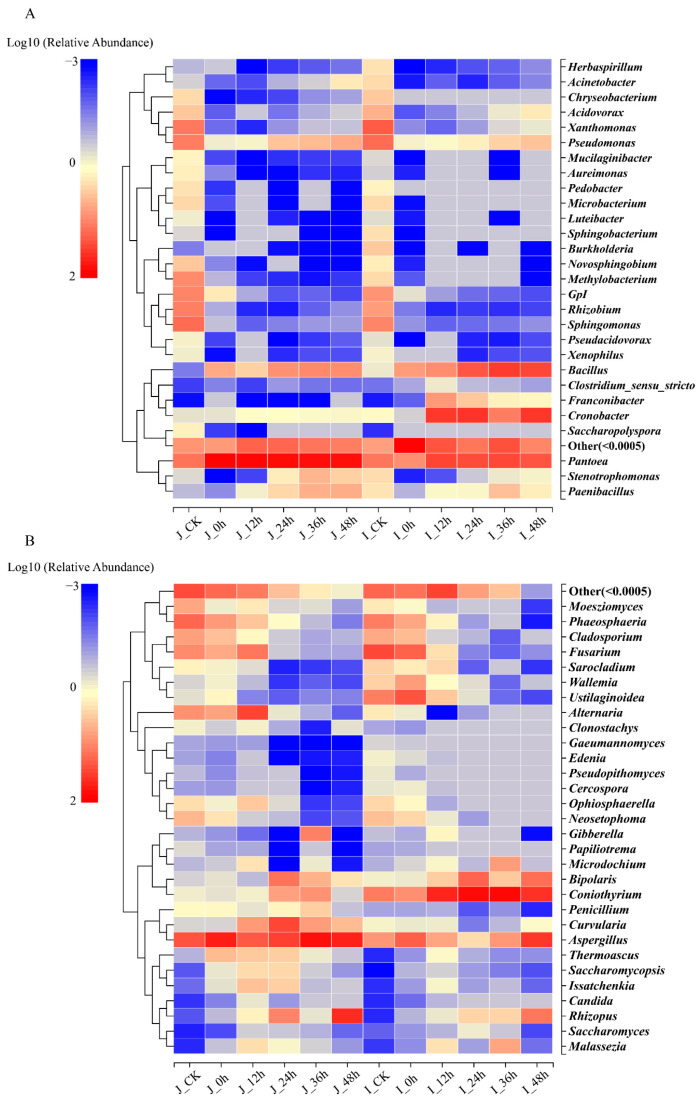
Heatmap of the changes in the microbial communities of BR samples at various germination times: (**A**) bacteria; (**B**) fungi. Labels ‘J’ and ‘I’ in legend refer to HLJ2 and HN samples, respectively.

To compare the microbial composition and community dynamics of the two cultivars under different periods, a heatmap was constructed at the genera level (Figure 5). As shown in Figure 5, the dominant bacterial species were significantly different among the different periods during the BR germination. The relative abundance of *Sphingomonas*, *Xanthomonas*, and *Pseudomonas* decreased rapidly, whereas the abundance of *Pantoea* increased obviously at the germinated stages. Importantly, the relative abundance of *Bacillus* in the two cultivars and *Cronobacter* in the HN samples was significantly higher in the germinated periods than in the ungerminated samples (Figure 5A). For the fungi, the most abundant genera in the HN samples were *Aspergillus*, *Phaeosphaeria*, *Fusarium*, and *Alternaria* at the initial stage, and their abundance reduced markedly with the extension of the germination time, except for that of *Aspergillus*. In contrast, the abundance of *Coniothyrium* showed the trend of growth for the germinated stages and became the dominant genus (Figure 5B). It showed that the germination process remarkably influenced the bacterial and fungal colony structures.

#### 3.3.5. LDA Effect Size (LEfSe) Analysis

**Figure 6:**
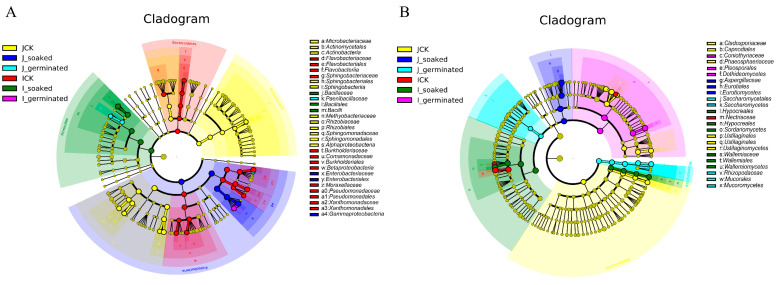
Landscape of bacterial (**A**) and fungal (**B**) clades in HLJ2 and HN samples. Control: untreated BR grains were used as the ungerminated samples. Soaked treatment: the BR kernels were soaked in distilled water at a ratio of 1:5 (*w*/*v*) at 30 °C for 12 h. Germinated treatment: the BR kernels were germinated at 30 °C for 12, 24, 36, and 48 h. Labels ‘J’ and ‘I’ in legend refer to HLJ2 and HN samples, respectively.

**Figure 7:**
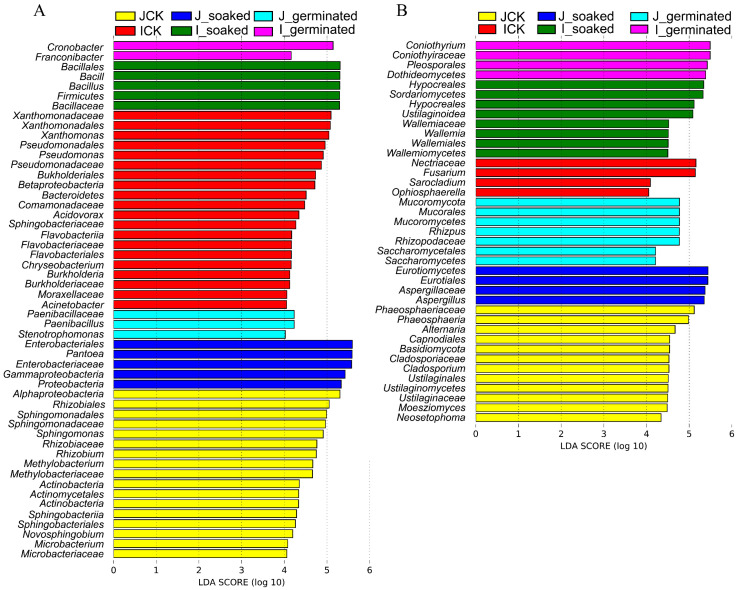
Differences in LEfSe in bacteria (**A**) and fungi (**B**) in HLJ2 and HN samples. Colored clades represent significant differences with an LDA threshold of 4.0. Control: untreated BR grains were used as the ungerminated samples. Soaked treatment: the BR kernels were soaked in distilled water at a ratio of 1:5 (*w*/*v*) at 30 °C for 12 h. Germinated treatment: the BR kernels were germinated at 30 °C for 12, 24, 36, and 48 h. Labels ‘J’ and ‘I’ in legend refer to HLJ2 and HN samples, respectively.

To understand the predominant microbiota composition at the different process stages (ungerminated, soaking, and germination), LEfSe was used to identify the bacterial and fungal taxa that were most likely to explain the differences between the HLJ2 and the HN samples (Figure 6 and Figure 7). There were in total 52 bacterial biomarkers with LDA scores over 4, indicating a significant influence of the sample treatments on the microbial species. As shown in Figure 6A, *Proteobacteria* and *Actinobacteria* dominated in the HLJ2 samples, while *Firmicutes* and *Bacteroidetes* dominated the HN samples. At the ungerminated stage, the bacteria significantly enriched in HLJ2 mainly belonged to *Rhizobium*, *Sphingomonas*, and *Methylobacterium*, while *Xanthomonas*, *Pseudomonas*, and *Acidovorax* were the main biomarkers in the HN samples. *Pantoea*, *Paenibacillus*, *Bacillus*, and *Cronobacter* were the most abundant genera in the HLJ2 and HN samples after soaking and germination, respectively (Figure 7A). For the fungi composition (Figure 6B and Figure 7B), 39 fungal biomarkers were significantly abundant with LDA scores over 4. *Ascomycota*, *Mucoromycota*, and *Basidiomycota* were the predominant phyla across the all of the stages. At the ungerminated stage, *Phaeosphaeria*, *Alternaria*, and *Cladosporium* were the marker genera in HLJ2, while *Fusarium* was the predominant genera in HN. *Aspergillus* and *Rhizopus* dominated in the HLJ2 samples, while *Ustilaginoidea*, *Wallemia*, and *Coniothyrium* dominated in the HN samples after soaking and germination, respectively.

**Figure 8:**
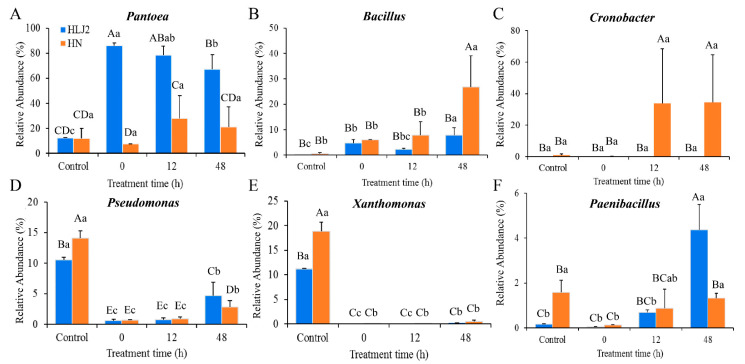
Bacterial genera difference analysis of BR samples at various germination times. (**A**) *Pantoea*; (**B**) *Bacillus*; (**C**) *Cronobacter*; (**D**) *Pseudomonas*; (**E**) *Xanthomonas*; (**F**) *Paenibacillus*. Different upper- and lower-case letters indicate that means were significantly different among the two samples and the different gemination stages for the same cultivar, respectively, at *p* = 0.05.

**Figure 9:**
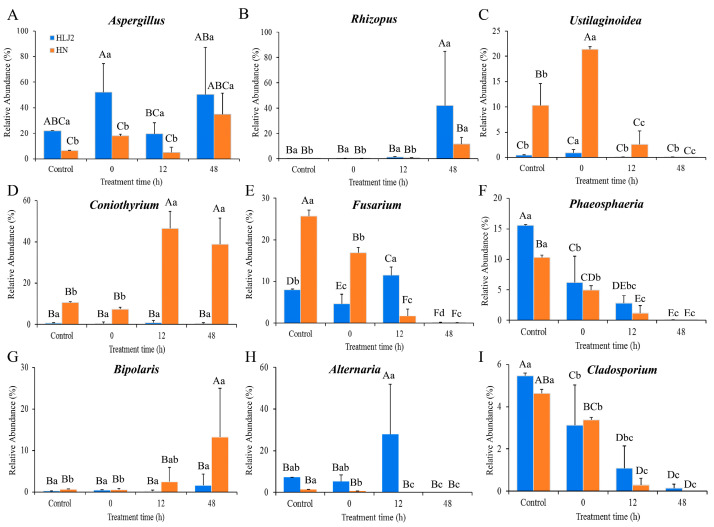
Difference analysis of fungal genera for BR samples at various germination times. (**A**) *Aspergillus*; (**B**) *Rhizopus*; (**C**) *Ustilaginoidea*; (**D**) *Coniothyrium*; (**E**) *Fusarium*; (**F**) *Phaeosphaeria*; (**G**) *Bipolaris*; (**H**) *Alternaria*; (**I**) *Cladosporium*. Different upper- and lower-case letters indicate that means were significantly different among two samples and the different germination stages for the same cultivar, respectively, at *p* = 0.05.

As shown in Figure 8, the relative abundance of *Pantoea*, *Paenibacillus*, and *Bacillus* increased, while the abundance of *Xanthomonas* and *Pseudomonas* decreased significantly in the HLJ2 samples after germination for 48 h (*p* < 0.05). In addition, at the end of the germination stage, the relative abundance of *Pantoea*, *Pseudomonas*, and *Paenibacillus* was higher in the HLJ2 than in the HN samples, while the changes in *Bacillus* and *Cronobacter* were reversed. In Figure 9, compared to the ungerminated samples, the relative abundance of *Aspergillus* and *Rhizopus* increased, while the abundance of *Fusarium*, *Phaeosphaeria*, and *Cladosporium* decreased significantly in the HLJ2 samples after germination for 48 h (*p* < 0.05). For the HN samples, significant differences in the relative abundance of nine genera were detected after being germinated for 48 h in comparison to the ungerminated samples. It was also found that the abundance of *Coniothyrium* and *Bipolaris* was lower in the HLJ2 than in the HN samples, while the abundance of *Rhizopus* was reversed (*p* < 0.05). Additionally, variations between the individual samples may affect the results (e.g., *Cronobacter*, *Rhizopus*, and *Alternaria*), requiring more replicates to eliminate.

#### 3.3.6. Functional Prediction Analysis of Microbial Community

**Figure 10:**
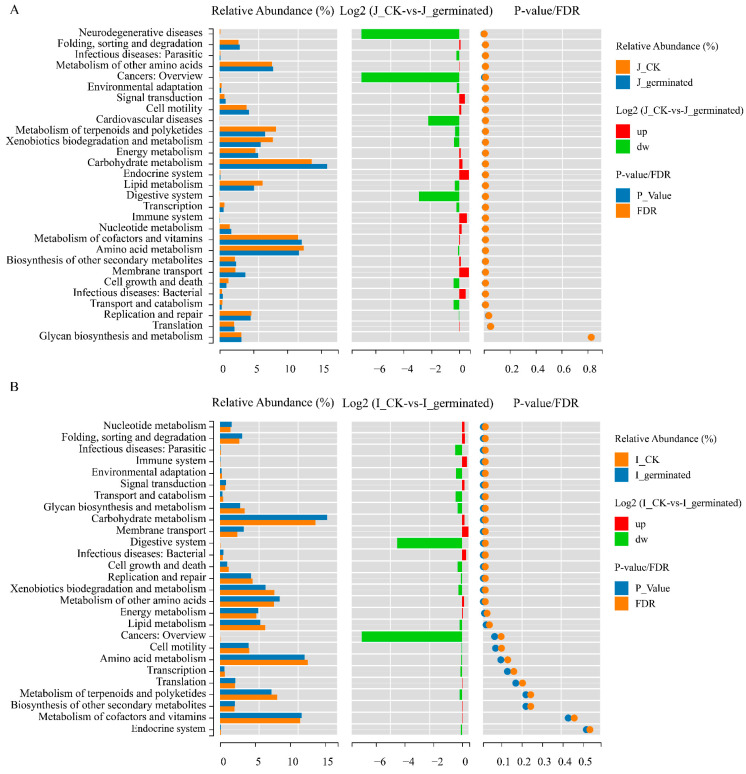
Differences in the functional properties of KEGG level 2 between ungerminated and germinated BR samples: (**A**) HLJ2, (**B**) HN. The pathway was significantly different between the two groups with *p*-value/FDR < 0.05. Labels ‘J’ and ‘I’ in legend refer to HLJ2 and HN samples, respectively.

PICRUSt was used to predict the functional genes of the microbial communities during the process of BR germination in this study. The relative abundance of the level 1 (Supplementary Figure S4A) and 2 (Figure S4B) functions of the bacterial microbes in KEGG was predicted based on the 16S rRNA gene sequence. As shown in Figure 10, the KEGG prediction of the function classification was essentially similar to that between the HLJ2 and HN samples during the different periods. The largest proportion in the all samples was the metabolism class, followed by genetic information processing (level 1), indicating that metabolism was the primary function of the bacterial community. Using the level 2 KEGG ortholog function predictions, 27 different functional categories between the HLJ2 and HN samples during BR germination were determined. Among the genes related to metabolism, they mainly included those of carbohydrate, amino acid, lipid, cofactor, vitamin, terpenoid, and polyketide metabolism (level 2). 

To further analyze the differences in these functions between the ungerminated and the germinated samples in HLJ2 and HN, the Wilcox test was used in the study (Figure 10). Using the level 2 KEGG function predictions, 27 significantly different functional categories between two groups in HLJ2 were observed (Figure 10A). The functional categories associated with the microbiota in the germinated samples compared to those in the ungerminated samples were significantly enriched, including carbohydrate metabolism, cofactor and vitamin metabolism, energy metabolism, membrane transport, and cell motility, whereas the abundance of functional genes in terpenoid and polyketide metabolism, transcription, amino acid metabolism, replication, and repair was significantly lower after germination. There were also significant changes in the functional classification of the HN samples, such as carbohydrate metabolism, xenobiotics biodegradation and metabolism, energy metabolism, membrane transport, and lipid metabolism (Figure 10B). These results indicated that the functional categories associated with the microbiota in the HLJ2 and HN samples changed significantly during the process of germination. It was consistent with the changes in the bacterial community during the BR germination process. In addition, the result also indicated that the functional categories of infectious disease performed the difference after germination. It is related to the potential involvement of the pathogenic microbial species in human diseases.

## 4. Discussion

In this study, we used the HTS technology to examine the microbial diversity and the dominant genera changes at different BR germination stages. The results indicated that the germination process significantly influenced the microbial composition and reduced the microbial diversity. The relative abundance of the dominant genera increased obviously during the BR germination. For the bacteria, the dominant genera of bacteria were *Pantoea*, *Bacillus*, and *Cronobacter*. For the fungi, *Aspergillus*, *Rhizopus*, and *Coniothyrium* increased obviously during germination. Previous studies also reported that the bacterial diversity of the seeds (alfalfa, radish, and rapeseed) and the fungal diversity of the barley seeds sharply declined after germination [8,20]. However, there were significant differences in the main dominant genera during the germination of the different seeds, which was due to the environmental factor and the different food products markedly affecting the microbial composition [20,28]. 

The high nutritional values of sprouts, along with the suitable warm and humid environments, could make them more susceptible to microbial contamination [29]. Previous studies have suggested that the majority of sprout-related foodborne disease has been related to contaminated seeds, and the initial microbes in seeds have important effects on the microbial community structure during germination [30,31]. The microbial contamination of the seeds depends on the environmental conditions, including pre-harvest practices and postharvest handling or processing [7]. In this study, the microbiological profiles of 13 BR samples from ten provinces in China were investigated. The results were consistent with the study reported by Kim et al. [4] that the counts of total mesophilic aerobic bacteria, yeasts and molds, and coliforms were 2.4 × 10^6^, 4.8 × 10^3^, and 4.2 × 10^3^ CFU/g, respectively. Park et al. [32] also showed that aerobic bacteria, as well as yeasts and molds, were found to be the predominant contaminants in BR grains, with the levels of 6.24 ± 0.02 and 6.16 ± 0.04 log CFU/g. *B. cereus* is a widespread endospore-forming pathogen that contaminates common grains and can be detected, with the exception of HN2 in this study. Kim et al. [33] investigated the contamination of rough rice collected from 64 farms in 22 agricultural areas, and the results showed that the rice samples were broadly contaminated with *B. cereus* spores. In addition, the preliminary results showed that Salmonella and *Listeria monocytogenes* were not detected in the BR samples (the detection limit was 1.30 log CFU/mL). Moreover, *L. monocytogenes* and *Escherichia coli* were not found during BR germination, while *Salmonella* with the low relative abundance (<0.05%) was observed by HTS analysis. This may be related to the variety of samples, sources, and handling.

Soaking is one of the important steps in GBR production. There are various biochemical reactions during sprouting, such as the conversion of starch, fat, and proteins into simpler forms, including glucose, fatty acids, and amino acids [34]. Therefore, the microbial counts significantly increased due to the favorable growth conditions in the soaking and sprouting process (Figure 1). In a previous study, the aerobic bacteria and the yeast and mold counts between BR and GBR were shown to increase from the initial 2.4 × 10^6^ to 3.0 × 10^7^ CFU/g and from 4.8 × 10^3^ to 1.7 × 10^5^ CFU/g [4]. However, there was no significant difference in the TVC and yeast/mold counts during the purple BR germination reported by Bourneow and Toontam [6]. The difference could be due to the variations in the source and cultivar of the samples and the sprouting conditions [6,8]. The initial microbial populations of the grains and the medium types could also contribute to the variations in the results [4]. Furthermore, several studies have found higher populations of microbes in sprouts compared with those of ungerminated seeds, including barley, alfalfa, radish, and rapeseed [8,20,35].

From the microbial alpha diversity analysis (Supplementary Tables S1 and S2), the richness and diversity of the two BR cultivars decreased significantly after germination, which is consistent with the results of Kim et al. [20]. However, there was no significant difference in microbial diversity with the extension of the germination time. In previous studies, it was also observed that the microbial diversity of the seeds was higher than that of the sprout products [36,37]. These changes may be due to the proliferation of the *Enterobacteriaceae* in the bacteria and the *Aspergillaceae* or *Coniothyriaceae* in the fungi during germination; then, the specific genera became dominant, caused an unbalanced community composition, and reduced the microbial diversity [20]. However, Ostlie et al. [8] observed that the bacterial diversity of barley increased significantly after germination. This may be related to the microbial structure of the grains and the variation of the treatment conditions. These results suggested that the cultivar of seeds affected the microbial diversity and composition, which is consistent with the previous reports [17,20,38]. 

The bacterial community composition of the BR samples was significantly influenced by the germination process (from seed to sprout) and the grain cultivars. At the phylum level, the bacterial community remained similar between the HLJ2 and HN samples during germination; however, the relative abundance of the predominant phyla differed at the various germinated stages. *Proteobacteria* was the dominant phyla at the all stages, but the abundance of *Firmicutes* increased with the extension in germination time. *Proteobacteria*, *Firmicutes*, *Actinobacteria*, and *Bacteroidetes* have been reported as the most abundant phyla in the soil and field in previous reports [39,40], indicating that these bacteria are widely distributed in the natural environment. Similar bacterial composition, but with a different relative abundance of the two cultivars before germination, was observed. This is consistent with the results reported by Ostlie et al. [8] and Kim et al. [17], in which the similar genera were detected in barley, radish, rapeseed, and alfalfa seeds. Notably, *Pantoea* was the most abundant genus in the HLJ2 samples, while *Cronobacter*, *Bacillus*, and *Pantoea* were dominant in the HN samples during germination (Figure 3A–C). *Pantoea* is a diverse group of rod-shaped Gram-negative bacteria in the *Enterobacteriaceae* [41], which can be confirmed by the culture-dependent results with the high population during germination (Figure 1). It is also a potential candidate of the spoilage-causing genus [17]. Previous studies reported that *Pantoea* was highly abundant in fresh mung bean sprouts, spinach [17,42], and barley [8], and several species were generally recognized as plant pathogens, but some species can cause disease in humans [41].

In this study, *Bacillus* (in HLJ2 and HN) and *Cronobacter* (in HN) were also the most abundant genera during germination. *Bacillus* species are fundamental to food fermentation, and several spoilage species and pathogens may cause food contamination and poisoning [15,43]. It has been reported that *Bacillus* is widely distributed in soil and water and can be detected with high abundance in radish, rapeseed, and alfalfa sprouts [20,28]. *Cronobacter* species are also commonly found in the environment or in soil and water [44], as well as in various food products, such as rice and other cereal seeds, vegetables, and fruits [45]. These bacteria are opportunistic pathogens associated with life-threatening infections in newborns [46]. *Pseudomonas* and *Lactococcus* were the most common bacterial species in the barley, alfalfa and mung sprouts [8,17], and fermented vegetables [47,48]. However, it was not the major species found in this study and reported by Kim et al. [20]. It was obvious that variations in the environmental factors and food products markedly affected the microbial composition. Due to the high nutritional values and suitable humid environment during the soaking and germination process, high abundance species, such as the *Pantoea* species, are stronger environmental competitors than the less competitive bacteria, leading to an unbalanced community composition [49]. Here, the growth of the *Pantoea*, *Bacillus*, *Cronobacter*, and other species influenced the bacterial community and consequently reduced the diversity indices. Furthermore, differences in the microbial communities between the HLJ2 and HN samples were attributed to endophytes (specific bacteria), which can vary with the species and the age of the host [50]. Therefore, two cultivars of BR may host differential bacterial species with differential functional profiles [20].

As with the bacteria, the fungal composition of the BR samples was also significantly influenced by the germination process and the cultivar of grain. Here, four known phyla were identified, and the most abundant phyla were *Ascomycota* and *Mucoromycota*, which agreed with the study results reported by Huang et al. [51] At the genus level, the top five genera (relative abundance > 5%) were *Aspergillus*, *Phaeosphaeria*, *Fusarium*, *Alternaria*, and *Cladosporium* in the ungerminated HLJ2 samples, while *Fusarium*, *Coniothyrium*, *Phaeosphaeria*, *Ustilaginoidea*, and *Aspergillus* dominated in the HN samples. It differed from the report by Ostlie et al. [6] that the most abundant fungi were *Cryptococcus*, *Cladosporium*, *Pyrenophora*, *Vagicola*, and *Sporobolomyces* in both ungerminated and germinated barley. The variation of the different grain endophytes may be one of the most important reasons for the difference in the microbial community [51]. During germination, the relative abundance of *Aspergillus* and *Rhizopus* in the HLJ2 samples rose, while *Coniothyrium*, *Aspergillus*, and *Rhizopus* increased significantly in the HN samples. Many reports have shown that the *Aspergillus* species as predominant fungi can be found in various agricultural products [52,53]. Our results agreed with Huang et al. [37] who found high frequencies of *Aspergillus* species in wheat seeds and sprouts. The microbiological analysis demonstrated that *Coniothyrium* and *Rhizopus* were predominant in a bean sauce mash, a traditional fermented soybean product [54]. Among these fungi, those such as the *Aspergillus* and *Fusarium* species can produce mycotoxins. Fungal growth caused the loss of nutritive substances and could also result in contamination by mycotoxins, which may be harmful to humans [55]. 

In addition, it was noteworthy that the relative abundance of *Fusarium* (HN), *Phaeosphaeria* (HLJ2 and HN), *Cladosporium* (HLJ2 and HN), and *Ustilaginoidea* (HN) decreased significantly during germination (Figure 9). *Fusarium*, *Cladosporium*, and *Alternaria* are considered as the most ubiquitous fungi on cereal grains and usually continue to survive at all stages of plant growth [56]. Ostlie et al. [8] observed that the relative abundance of *Fusarium* and *Alternaria* increased and *Cladosporium* significantly decreased after the germination of barley. Most *Phaeosphaeria* species are plant pathogens, which can cause serious infections, especially in many important crops, such as wheat and corn [57]. The previous study showed that seed endophytes may be important founders of the sprout microbial communities. In the work, the bacterial and fungal community in ungerminated BR grains is similar to that in the germinated samples; so, some bacteria and fungi derived from grains were transferred into the sprouts. 

The predictive functions of the bacterial community were further analyzed using PICRUSt based on the KEGG database in this study. It is notable that each sample performed the similar gene function with a different abundance (Supplementary Figure S4), which may be due to the similarity in gene functions of the different microbiota [58]. The predicted bacterial functional profiles exhibited the highest relative abundance in metabolism, particularly the carbohydrate, amino acid, lipid, cofactor, vitamin, terpenoid, and polyketide metabolisms. These metabolic pathways are crucial for supporting microbial growth. According to the Wilcox test, the functional categories associated with the microbiota in the germinated samples compared to those in the ungerminated samples were significantly different (Figure 10). Carbohydrate metabolism and amino acid metabolism are essential for microbes, which play an important role in the survival of microorganisms and their related functions [59]. Moreover, the relative abundance of membrane transport and energy metabolism was higher in the germinated stage compared with the ungerminated stage. These results suggest that the changes in bacterial composition during BR germination may be related to differences in the functional metabolism of the microbiota. The detection of *Bacillus* and *Cronobacter* in BR during germination highlights the potential risk of the foodborne illness associated with the consumption of sprouts. In this study, PICRUSt identified the metabolic pathways associated with infectious diseases, supporting the potential involvement of the identified microbial species in human diseases (Figure 10). These findings suggest that sprouted BR may pose a safety hazard to consumers.

This study showed that microbial diversity and composition were affected by the BR cultivars. Despite the selected BR samples being the most consumed varieties, it is necessary to investigate the microbial diversity of more diversified varieties. The associations between the changes in nutrient composition and microbiota of BR during germination also needs to be further explored. In addition to the pathogenic and potential pathogenic genera, the information on the several spoilage genera associated with sprouts also requires attention. In addition, it is important to consider sterilization before the preparation for the BR germination and the optimal process conditions, especially temperature control during incubation.

## 5. Conclusions

In this study, both microbial cultivation and HTS technology were developed to explore the microbial community dynamics of BR during germination. The microbial counts of the BR samples rose significantly with the prolongation of the germination time. The results indicated that germination significantly influenced the microbial composition and diversity. Different microbial richness but similar microbial community diversity was found between the HLJ2 and HN samples. During germination, *Proteobacteria* and *Firmicutes* were the predominant phyla, while *Pantoea*, *Bacillus*, and *Cronobacter* were the key microbial groups at the genus level. For the fungi, *Aspergillus*, *Rhizopus*, and *Coniothyrium* were the dominant genera in the HLJ2 and HN samples. The abundance of several pathogenic and spoilage microorganisms carried in the BR seeds increased significantly after germination treatment, thereby causing the risk of foodborne illness. These findings suggest that microbial community dynamics analysis can provide related information to establish control points during BR germination and can be useful in ensuring microbial safety in the industry.

## Figures and Tables

**Table 1:** Microbial counts of 13 BR samples.

Samples	Cultivars	Producing Regions	Moisture Content (% w.b.)	Viable Counts (log10 CFU/g)			
				TVC	Molds and Yeasts	*Bacillus cereus*	*Enterobacteriaceae*
AH1	Japonica	Anhui	12.21 ± 0.32	5.45 ± 0.20 ^abcd^	4.11 ± 0.16 ^c^	<1.3	3.38 ± 0.34 ^e^
AH2	Japonica	Anhui	11.99 ± 0.10	5.11 ± 0.24 ^d^	4.22 ± 0.15 ^bc^	1.60 ± 0.49 ^e^	3.91 ± 0.33 ^d^
GD	Indica	Guangdong	12.12 ± 0.35	5.79 ± 0.05 ^a^	5.33 ± 0.28 ^a^	2.45 ± 0.17 ^ab^	4.76 ± 0.25 ^b^
GX	Japonica	Guangxi	14.00 ± 0.33	4.10 ± 0.11 ^f^	5.20 ± 0.14 ^a^	1.76 ± 0.11 d ^e^	3.09 ± 0.09 ^e^
HB	Indica	Hubei	11.89 ± 0.22	4.55 ± 0.10 ^e^	4.22 ± 0.09 ^bc^	2.59 ± 0.16 ^a^	3.17 ± 0.06 ^e^
HLJ1	Indica	Heilongjiang	11.61 ± 0.91	4.41 ± 0.09 ^ef^	4.16 ± 0.45 ^bc^	<1.3	3.86 ± 0.52 ^d^
HLJ2	Japonica	Heilongjiang	11.42 ± 0.66	5.32 ± 0.28 ^bcd^	4.60 ± 0.45 ^abc^	2.05 ± 0.34 ^bcd^	3.98 ± 0.33 ^cd^
HN	Indica	Hunan	12.53 ± 0.24	5.18 ± 0.02 ^cd^	4.65 ± 0.16 ^abc^	2.30 ± 0.32 ^abc^	3.94 ± 0.35 ^d^
JS	Japonica	Jiangsu	12.75 ± 0.29	5.44 ± 0.29 ^abcd^	5.00 ± 0.26 ^a^	1.54 ± 0.34 ^e^	4.37 ± 0.08 ^c^
JX	Japonica	Jiangxi	13.55 ± 0.14	5.11 ± 0.19 ^d^	4.86±0.51 ^ab^	<1.3	3.33 ± 0.24 ^e^
LN1	Japonica	Liaoning	11.84 ± 0.05	5.86 ± 0.13 ^a^	5.14±0.39 ^a^	1.99 ± 0.24 ^dce^	5.22 ± 0.23 ^a^
LN2	Japonica	Liaoning	12.43 ± 0.05	5.60 ± 0.01 ^abc^	3.93±0.03 ^c^	ND *	3.34 ± 0.26 ^e^
SC	Japonica	Sichuan	11.67 ± 0.25	5.65 ± 0.22 ^ab^	4.98±0.26 ^a^	1.61 ± 0.32 ^e^	4.13 ± 0.09 ^cd^

* ND = Not detected because below the limit of 1.30 log CFU/g. Values with different lowercase letters in the same column showed significant differences at *p* < 0.05.

## Data Availability

The data presented in this study are available on request from the corresponding author.

## Supplementary Materials

The following supporting information can be downloaded at: https://www.mdpi.com/article/10.3390/foods12040755/s1, Figure S1: rarefaction curves of bacterial (A) and fungal (B) populations in the germination process of BR samples, Figure S2: Venn diagrams for numbers of shared and unique genera of ungerminated BR samples in bacteria (A) and fungi (B), Figure S3: flower diagrams for numbers of shared and unique genera of BR samples in bacteria (A,B) and fungi (C,D) at various germination times; (A,C) HLJ2; (B,D) HN, Figure S4: relative abundance of functional properties of KEGG levels 1 (A) and 2 (B) based on the 16S rRNA gene sequences using PICRUSt, Table S1: richness and diversity indices for bacteria of BR samples during germination, Table S2: richness and diversity indices for fungi of BR samples during germination.

## References

[1] Saleh A.S.M., Wang P., Wang N., Yang L., Xiao Z. (2019). Brown rice versus white rice: Nutritional quality, potential health benefits, development of food products, and preservation technologies. Compr. Rev. Food Sci. Food Saf..

[2] Lemmens E., Moroni A.V., Pagand J., Heirbaut P., Ritala A., Karlen Y., Lê K.A., Den Broeck H.C., Brouns F.J.P.H., Brier N. (2019). Impact of cereal seed sprouting on its nutritional and technological properties: A critical review. Compr. Rev. Food Sci. Food Saf..

[3] Cho D.H., Lim S.T. (2016). Germinated brown rice and its bio-functional compounds. Food Chem..

[4] Kim K.S., Kim B.H., Kim M.J., Han J.K., Kum J.S., Lee H.Y. (2012). Quantitative microbiological profiles of brown rice and germinated brown rice. Food Sci. Biotechnol..

[5] Lu Z.H., Zhang Y., Li L.T., Curtis R.B., Kong X.L., Fulcher R.G., Zhang G., Cao W. (2010). Inhibition of microbial growth and enrichment of gamma-aminobutyric acid during germination of brown rice by electrolyzed oxidizing water. J. Food Protect..

[6] Bourneow C., Toontam N. (2019). Microbiological quality and some bioactive compounds in relation to sensory profiles during germination of brown-purple-pigmented rice. Int. Food Res. J..

[7] Miyahira R.F., Costa Antunes A.E. (2021). Bacteriological safety of sprouts: A brief review. Int. J. Food Microbiol..

[8] Ostlie H.M., Porcellato D., Kvam G., Wicklund T. (2021). Investigation of the microbiota associated with ungerminated and germinated Norwegian barley cultivars with focus on lactic acid bacteria. Int. J. Food Microbiol..

[9] Los A., Ziuzina D., Boehm D., Cullen P.J., Bourke P. (2017). The potential of atmospheric air cold plasma for control of bacterial contaminants relevant to cereal grain production. Innov. Food Sci. Emerg. Technol..

[10] Laca A., Mousia Z., Diaz M., Webb C., Pandiella S.S. (2006). Distribution of microbial contamination within cereal grains. J. Food Eng..

[11] Piernas V., Guiraud J.P. (1997). Microbial hazards related to rice sprouting. Int. J. Food Sci. Technol..

[12] Dechet A.M., Herman K.M., Chen Parker C., Taormina P., Johanson J., Tauxe R.V., Mahon B.E. (2014). Outbreaks caused by sprouts, United States, 1998–2010: Lessons learned and solutions needed. Foodborne Pathog. Dis..

[13] Callejón R.M., Rodriguez-Naranjo M.I., Ubeda C., Hornedo-Ortega R., GarciaParrilla M.C., Troncoso A.M. (2015). Reported foodborne outbreaks due to fresh produce in the United States and European union: Trends and causes. Foodborne Pathog. Dis..

[14] Villeneuve S., Power K.A., Guévremont E., Mondor M., Tsao R., Wanasundara J.P.D., Mercier S. (2015). Effect of a short-time germination process on the nutrient composition, microbial counts and bread-making potential of whole flaxseed. J. Food Process. Pres..

[15] Cai H., Zhang T., Zhang Q., Luo J., Cai C., Mao J. (2018). Microbial diversity and chemical analysis of the starters used in traditional Chinese sweet rice wine. Food Microbiol..

[16] Zhao G., Li J., Zheng F., Yao Y. (2021). The fermentation properties and microbial diversity of soy sauce fermented by germinated soybean. J. Sci. Food Agric..

[17] Keshri J., Krouptiski Y., Abu-Fani L., Achmon Y., Bauer T.S., Zarka O., Maler I., Pinto R., Saldinger S.S. (2019). Dynamics of bacterial communities in alfalfa and mung bean sprouts during refrigerated conditions. Food Microbiol..

[18] Los A., Ziuzina D., Boehm D., Bourke P. (2020). Effects of cold plasma on wheat grain microbiome and antimicrobial efficacy against challenge pathogens and their resistance. Int. J. Food Microbiol..

[19] Solanki M.K., Abdelfattah A., Britzi M., Zakin V., Wisniewski M., Droby S., Sionov E. (2019). Shifts in the composition of the microbiota of stored wheat grains in response to fumigation. Front. Microbiol..

[20] Kim S.Y., Ban G.H., Hong Y.W., Jang M.J., Kim S.A. (2022). Microbiome shifts in sprouts (alfalfa, radish, and rapeseed) during production from seed to sprout using 16S rRNA microbiome sequencing. Food Res. Int..

[21] Moongngarm A., Saetung N. (2010). Comparison of chemical compositions and bioactive compounds of germinated rough rice and brown rice. Food Chem..

[22] Magoc T., Salzberg S.L. (2011). FLASH: Fast length adjustment of short reads to improve genome assemblies. Bioinformatics.

[23] Edgar R.C. (2013). UPARSE: Highly accurate OTU sequences from microbial amplicon reads. Nat. Methods.

[24] Edgar R.C., Haas B.J., Clemente J.C., Quince C., Knight R. (2011). UCHIME improves sensitivity and speed of chimera detection. Bioinformatics.

[25] Caporaso J.G., Kuczynski J., Stombaugh J., Bittinger K., Bushman F.D., Costello E.K., Fierer N., Pena A.G., Goodrich J.K., Gordon J.I. (2010). QIIME allows analysis of high-throughput community sequencing data. Nat. Methods.

[26] Edgar R.C. (2010). Search and clustering orders of magnitude faster than BLAST. Bioinformatics.

[27] Schloss P.D., Westcott S.L., Ryabin T., Hall J.R., Hartmann M., Hollister E.B., Lesniewski R.A., Oakley B.B., Parks D.H., Robinson C.J. (2009). Introducing mothur: Open-source, platform-independent, community-supported software for describing and comparing microbial communities. Appl. Environ. Microbiol..

[28] Landry K.S., Sela D.A., Mclandsborough L. (2017). Influence of sprouting environment on the microbiota of sprouts. J. Food Saf..

[29] Liu H.K., Li Z.H., Zhang X.W., Liu Y.P., Zhao X.Y. (2021). The effects of ultrasound on the growth, nutritional quality and microbiological quality of sprouts. Trends Food Sci. Technol..

[30] Landry K.S., Micheli S., Mcclements D.J., Mclandsborough L. (2015). Effectiveness of a spontaneous carvacrol nanoemulsion against *Salmonella* enterica Enteritidis and *Escherichia coli* O157:H7 on contaminated broccoli and radish seeds. Food Microbiol..

[31] Peñas E., Gómez R., Frías J., Vidal-Valverde C. (2010). Effects of combined treatments of high pressure, temperature and antimicrobial products on germination of mung bean seeds and microbial quality of sprouts. Food Control.

[32] Park H., Puligundla P., Mok C. (2020). Cold plasma decontamination of brown rice: Impact on biochemical and sensory qualities of their corresponding seedlings and aqueous tea infusions. LWT Food Sci. Technol..

[33] Kim B., Bang J., Kim H., Kim Y., Kim B.S., Beuchat L.R., Ryu J.H. (2014). *Bacillus cereus* and *Bacillus thuringiensis* spores in Korean rice: Prevalence and toxin production as affected by production area and degree of milling. Food Microbiol..

[34] Mir S.A., Farooq S., Shah M.A., Sofi S.A., Khaneghah A.M. (2021). An overview of sprouts nutritional properties, pathogens and decontamination technologies. LWT Food Sci. Technol..

[35] Li X., Cai G., Wu D., Zhang M., Lin C., Lu J. (2018). Microbial community dynamics of Dan’er barley grain during the industrial malting process. Food Microbiol..

[36] Gloria T.C., Sophie B., Olivier B., Clémence G., Martial B., Marie-Agnès J., Matthieu B. (2018). Functional microbial features driving community assembly during seed germination and emergence. Front. Plant Sci..

[37] Huang Y., Kuang Z., Wang W., Cao L. (2016). Exploring potential bacterial and fungal biocontrol agents transmitted from seeds to sprouts of wheat. Biol. Control.

[38] Bziuk N., Maccario L., Straube B., Wehner G., Sorensen S.J., Schikora A., Small K. (2021). The treasure inside barley seeds: Microbial diversity and plant beneficial bacteria. Environ. Microbiome.

[39] Fierer N., Bradford M.A., Jackson R.B. (2007). Toward an ecological classification of soil bacteria. Ecology.

[40] He H.D., Li W.C., Yu R.Q., Ye Z.H. (2017). Illumina-based analysis of bulk and rhizosphere soil bacterial communities in paddy fields under mixed heavy metal contamination. Pedosphere.

[41] Walterson A.M., Stavrinides J. (2015). *Pantoea*: Insights into a highly versatile and diverse genus within the *Enterobacteriaceae*. FEMS Microbiol. Rev..

[42] Leff J.W., Noah F., Gabriele B. (2013). Bacterial communities associated with the surfaces of fresh fruits and vegetables. PLoS ONE.

[43] Logan N.A. (2011). *Bacillus* and relatives in foodborne illness. J. Appl. Microbiol..

[44] Chenu J.W., Cox J.M. (2010). *Cronobacter* (‘*Enterobacter sakazakii*’): Current status and future prospects. Lett. Appl. Microbiol..

[45] Berthold-Pluta A., Garbowska M., Stefańska I., Pluta A. (2017). Microbiological quality of selected ready-to-eat leaf vegetables, sprouts and non-pasteurized fresh fruit-vegetable juices including the presence of *Cronobacter* spp.. Food Microbiol..

[46] Healy B., Cooney S., O’Brien S., Iversen C., Whyte P., Nally J., Callanan J.J., Fanning S. (2010). *Cronobacter* (*Enterobacter sakazakii*): An opportunistic foodborne pathogen. Foodborne Pathog. Dis..

[47] He Z., Chen H., Wang X., Lin X., Ji C., Li S., Liang H. (2020). Effects of different temperatures on bacterial diversity and volatile flavor compounds during the fermentation of suancai, a traditional fermented vegetable food from northeastern China. LWT Food Sci. Technol..

[48] Zhang J., Zhang C., Wu W., Lv X., Xin X., Liu D., Hu H., Guo S. (2021). Correlation of the bacterial communities with umami components, and chemical characteristics in Zhejiang xuecai and fermented brine. Food Res. Int..

[49] Jiang L., Jeong J.C., Lee J.S., Park J.M., Yang J.W., Lee M.H., Choi S.H., Kim C.Y., Kim D.H., Kim S.W. (2019). Potential of *Pantoea dispersa* as an effective biocontrol agent for black rot in sweet potato. Sci. Rep..

[50] Singha K.M., Singh B., Pandey P. (2021). Host specific endophytic microbiome diversity and associated functions in three varieties of scented black rice are dependent on growth stage. Sci. Rep..

[51] Huang Y., Zhang M., Deng Z., Cao L. (2018). Evaluation of probiotic diversity from soybean (*Glycine max*) seeds and sprouts using illumina-based sequencing method. Probiotics Antimicrob. Proteins.

[52] Park H.S., Jun S.C., Han K.H., Hong S.B., Yu J.H. (2017). Diversity, application, and synthetic biology of industrially important *Aspergillus* Fungi. Adv. Appl. Microbiol..

[53] Perrone G., Susca A., Cozzi G., Ehrlich K., Varga J., Frisvad J.C., Meijer M., Noonim P., Mahakamchanakul W., Samson R.A. (2007). Biodiversity of *Aspergillus* species in some important agricultural products. Stud. Mycol..

[54] Xie M., An F., Wu J., Liu Y., Shi H., Wu R. (2019). Meta-omics reveal microbial assortments and key enzymes in bean sauce mash, a traditional fermented soybean product. J. Sci. Food Agric..

[55] Justé A., Malfliet S., Lenaerts M., Cooman L.D., Lievens B. (2011). Microflora during malting of barley: Overview and impact on malt quality. Brew. Sci..

[56] Gagkaeva T.Y., Gavrilova O.P., Orina A.S., Blinova E.V., Loskutov I.G. (2017). Response of wild Avena species to fungal infection of grain. Crop. J..

[57] El-Demerdash A. (2018). Chemical diversity and biological activities of *Phaeosphaeria* fungi genus: A systematic review. J. Fungi.

[58] Zheng X., Xu X., Ma Y., Zhu L., Xiao J., Deng L., Shi X., Wang B. (2021). Diversity and potential function of bacterial communities during milk fermentation of Kazak artisanal cheese. Process. Biochem..

[59] Wang Y., Cai W., Wang W., Shu N., Zhang Z., Hou Q., Shan C., Guo Z. (2021). Analysis of microbial diversity and functional differences in different types of high-temperature Daqu. Food Sci. Nutr..

