# Supplementary material for: Analysis of Dynamics and Diversity of Microbial Community during Production of Germinated Brown Rice
Source: Foods. 2023 Feb 9;12(4):755. doi: 10.3390/foods12040755 (PMC9956166; doi:10.3390/foods12040755)
Supplement: Supplementary file 1 [file foods-12-00755-s001.zip › foods-2149230-supplementary.pdf]

foods

MDPI

Article

Analysis of Dynamics and Diversity of Microbial Community during Production of Germinated Brown Rice

Gaoji Yang [1], Juanjuan Xu [1], Yuanmei Xu [1], Rui Li [1] and Shaojin Wang [1,2,*]

Supplementary Material

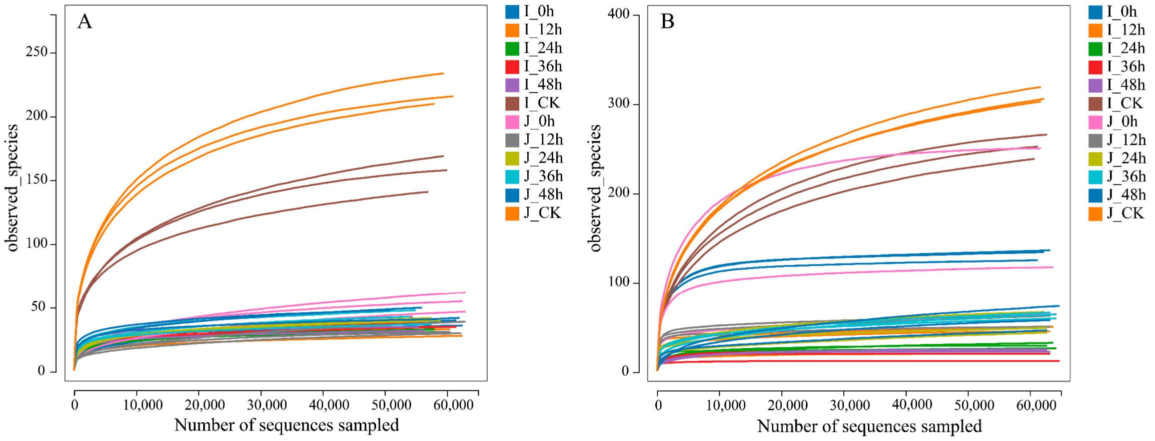

Figure S1. Rarefaction curves of bacterial (**A**) and fungal (**B**) populations in the germination process of BR samples. Control: untreated BR grains were used as the ungerminated samples. Samples germinated for 0 h represent the soaked BR. Labels 'J' and 'I' in legend refer to HLJ2 and HN samples, respectively.

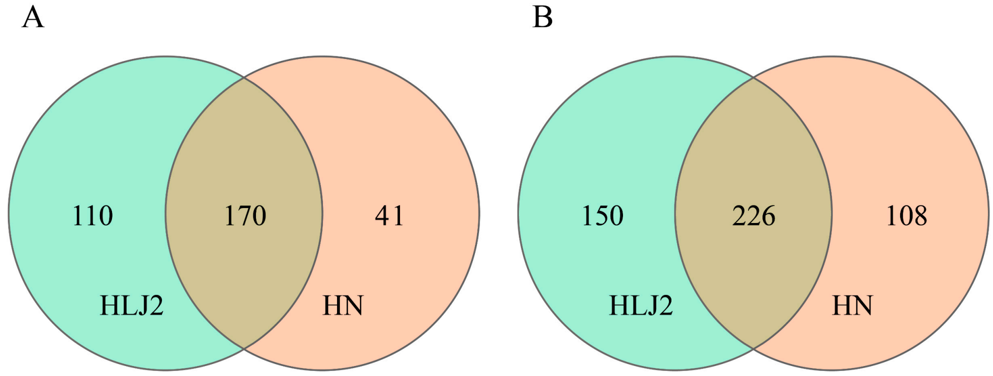

Figure S2. Venn diagrams for numbers of shared and unique genera of ungerminated BR samples in bacteria (**A**) and fungi (**B**).

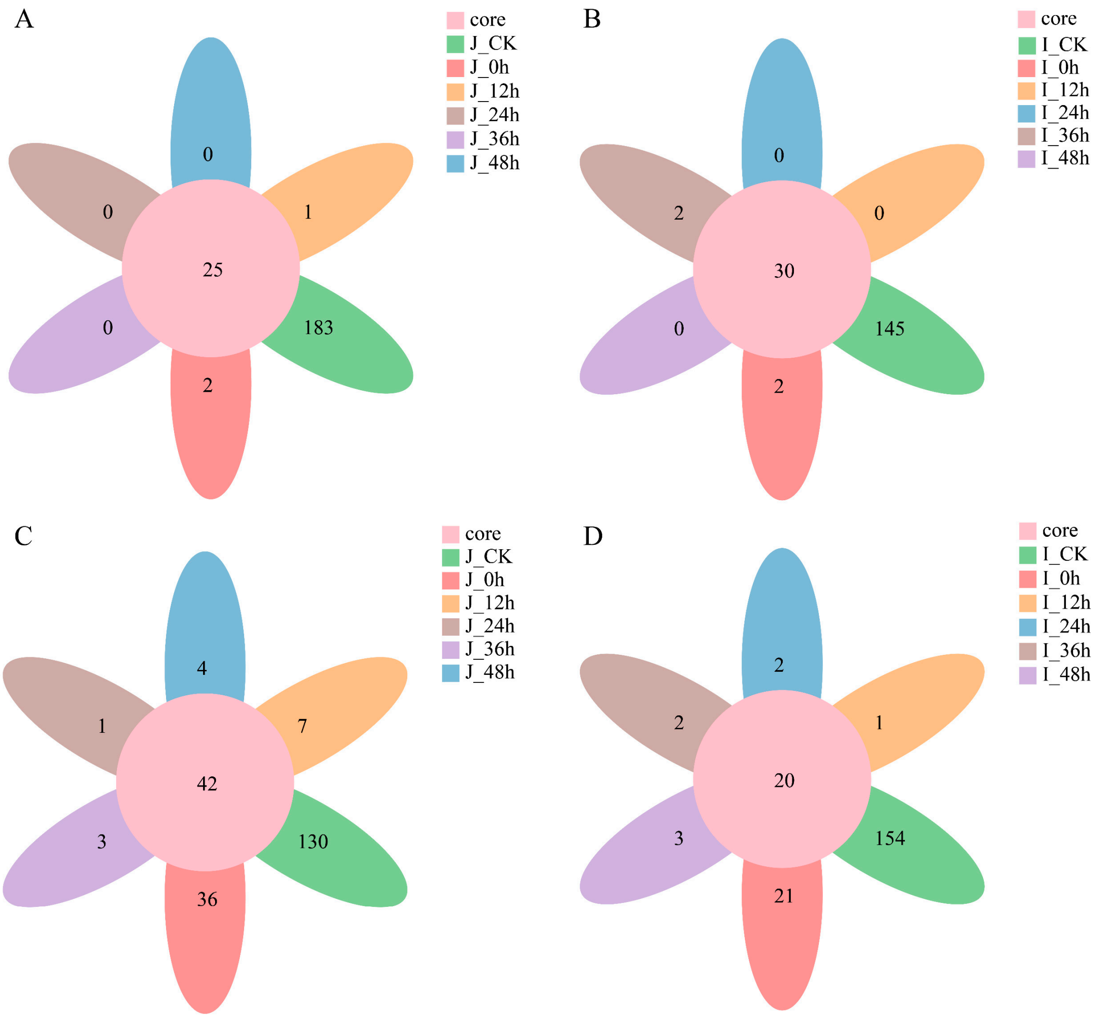

Figure S3. Flower diagrams for numbers of shared and unique genera of BR samples in bacteria (**A**,**B**) and fungi (**C**,**D**) at various germination times; (**A**,**C**) HLJ2; (**B**,**D**) HN. Labels 'J' and 'I' in legend refer to HLJ2 and HN samples, respectively.

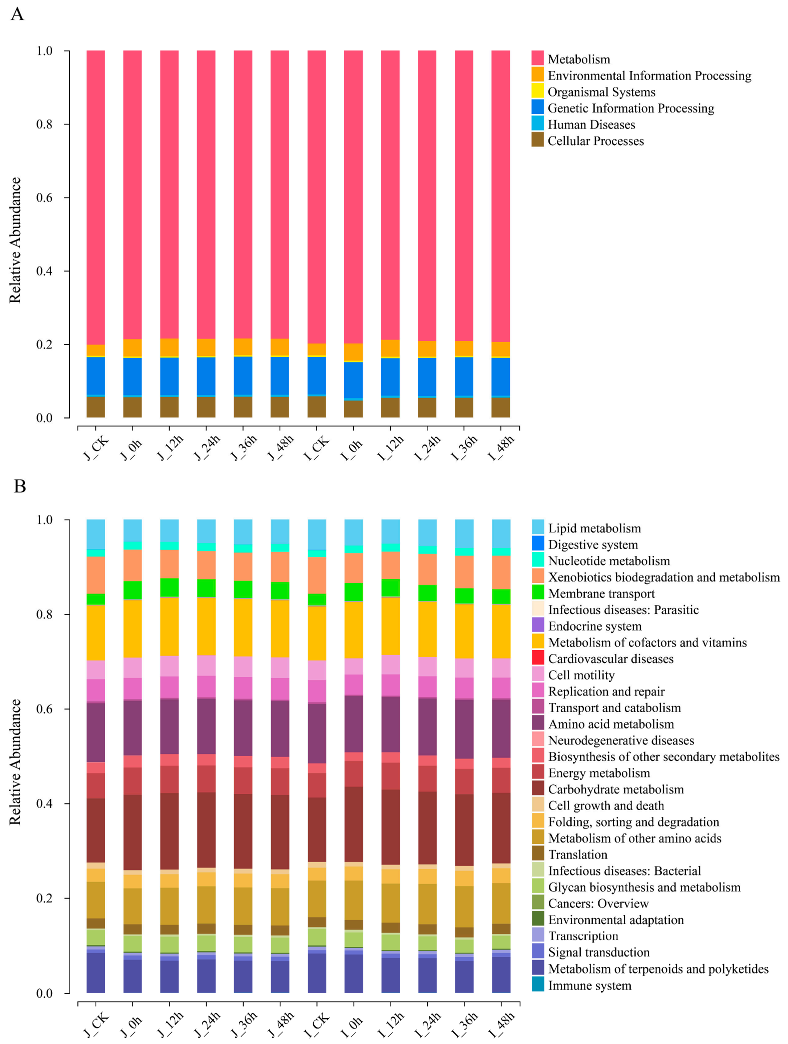

Figure S4. Relative abundance of functional properties of KEGG levels 1 (**A**) and 2 (**B**) based on the 16S rRNA gene sequences using PICRUSt. Labels 'J' and 'I' in legend refer to HLJ2 and HN samples, respectively.

Table S1. Richness and diversity indices for bacteria of BR samples during germination.

Samples	Germination Time (h)	Effective Tags	Sobs	Chao	ACE	Shannon	Simpson	Coverage (%)
HLJ2	Control	59,826 ± 1678	220.00 ± 12.49 A; a	235.89 ± 14.86 A; a	240.14 ± 17.27 A; a	3.14 ± 0.03 A; a	0.068 ± 0.002 E; b	99.95
	0	63,221 ± 286	54.67 ± 7.51 B; b	69.32 ± 15.26 C; b	81.32 ± 16.30 C; b	0.88 ± 0.10 AB; c	0.637 ± 0.042 E; a	99.98
	12	62,038 ± 1123	33.33 ± 4.93 D; c	42.12 ± 10.87 DEF; c	51.81 ± 18.03 CDE; b	1.00 ± 0.23 ABC; bc	0.548 ± 0.125 E; a	99.99
	24	58,154 ± 1086	40.67 ± 0.58 D; c	56.03 ± 8.64 DEF; c	63.75 ± 13.24 EF; b	1.32 ± 0.25 BCD; bc	0.451 ± 0.098 CDE; a	99.98
	36	55,790 ± 1581	42.67 ± 5.51 D; bc	49.37 ± 10.32 CD; bc	60.37 ± 11.56 DE; b	1.46 ± 0.25 DE; b	0.423 ± 0.137 ABCD; a	99.99
	48	55,406 ± 3897	46.33 ± 4.73 D; bc	51.37 ± 6.55 DE; bc	56.40 ± 5.00 CD; b	1.50 ± 0.42 F; b	0.437 ± 0.166 ABC; a	99.99
HN	Control	58,992 ± 1548	156.00 ± 14.11 B; a	171.09 ± 15.67 B; a	178.02 ± 18.63 B; a	3.01 ± 0.11 A; a	0.084 ± 0.011 E; c	99.96
	0	62,304 ± 603	39.33 ± 3.06 C; b	48.73 ± 9.34 C; b	61.03 ± 19.34 CD; b	0.70 ± 0.01 A; c	0.713 ± 0.002 E; a	99.98
	12	61,810 ± 992	32.00 ± 3.61 D; b	34.08 ± 4.26 EF; b	44.87 ± 9.25 EF; b	1.48 ± 0.28 CD; b	0.332 ± 0.120 DE; b	99.99
	24	58,295 ± 601	32.33 ± 2.00 D; b	37.58 ± 5.66 EF; b	38.08 ± 10.08 F; b	1.44 ± 0.30 F; b	0.343 ± 0.145 A; b	99.98
	36	59,158 ± 2437	36.33 ± 2.31 D; b	41.50 ± 8.26 F;b	43.36 ± 6.67 F; b	1.56 ± 0.08 F; b	0.283 ± 0.026 AB; b	99.99
	48	55,832 ± 1095	36.67 ± 5.69 D; b	41.11 ± 9.44 F;b	44.33 ± 7.37 F; b	1.61 ± 0.18 DE; b	0.298 ± 0.093BCDE; b	99.99

Different upper- and lower-case letters in the same column indicate that means were significantly different between two samples and different germination stage for each cultivar, respectively, at $p = 0.05$.

Table S2. Richness and diversity indices for fungi of BR samples during germination.

Samples	Germination Time (h)	Effective Tags	Sobs	Chao	ACE	Shannon	Simpson	Coverage (%)
HLJ2	Control	61,779 ± 433	309.33 ± 8.50 A; a	354.71 ± 10.75 A; a	362.58 ± 8.78 A; a	2.95 ± 0.01 A; a	0.095 ± 0.001 E; c	99.90
	0	63,339 ± 1191	144.00 ± 95.41 C; b	151.94 ± 87.86 C; b	174.47 ± 68.19 C; b	2.64 ± 0.59 AB; ab	0.180 ± 0.095 E; bc	99.98
	12	63,112 ± 192	59.00 ± 7.81 D; c	73.64 ± 23.25 DEF; bc	110.08 ± 74.65 CDE; bc	2.53 ± 0.37 ABC; ab	0.166 ± 0.092 E; bc	99.99
	24	62,656 ± 206	53.33 ± 12.10 D; c	61.17 ± 12.35 DEF; c	70.94 ± 19.29 EF; c	1.93 ± 0.81 BCD; bc	0.308 ± 0.279 CDE; abc	99.98
	36	64,065 ± 695	63.67 ± 3.51 D; c	109.17 ± 46.78 CD; bc	97.29 ± 10.62 DE; bc	1.32 ± 0.52 DE; cd	0.465 ± 0.229 ABCD; ab	99.97
	48	63,499 ± 1015	59.33 ± 14.05 D; c	90.48 ± 22.96 DE; bc	140.34 ± 59.61 CD; bc	0.92 ± 0.33 F; d	0.564 ± 0.168 ABC; a	99.97
HN	Control	61,555 ± 988	252.00 ± 13.00 B; a	275.54 ± 6.13 B; a	281.34 ± 4.92 B; a	3.04 ± 0.03 A; a	0.095 ± 0.002 E; c	99.93
	0	62,354 ± 912	132.00 ± 6.24 C; b	148.44 ± 24.69 C; b	144.53 ± 19.58 CD; b	3.06 ± 0.02 A; a	0.091 ± 0.001 E; c	99.99
	12	63,263 ± 663	38.67 ± 15.50 D; c	42.28 ± 19.51 EF; bc	49.51 ± 28.70 EF; bc	1.87 ± 0.40 CD; b	0.291 ± 0.068 DE; b	99.99
	24	63,617 ± 767	30.00 ± 2.00 D; cd	34.58 ± 4.13 EF; c	26.20 ± 24.36 F; c	0.85 ± 0.34 F; c	0.614 ± 0.190 A; a	99.99
	36	63,717 ± 1134	19.67 ± 5.13 D; d	19.67 ± 5.13 F; bc	19.87 ± 4.80 F; bc	0.92 ± 0.35 F; c	0.587 ± 0.148 AB; a	100.00
	48	62,761 ± 272	25.00 ± 1.00 D; cd	25.33 ± 0.58 F; bc	25.55 ± 0.80 F; bc	1.29 ± 0.18 DE; c	0.339 ± 0.068BCDE; b	100.00

One-way ANOVA was also applied as in Table S2. Different upper- and lower-case letters in the same column indicated that means were significantly different between the two samples and different germination stage for each cultivar, respectively, at $p = 0.05$.
